# Association of high body mass index, waist circumference, and body fat percentage with sarcopenia in older women

**DOI:** 10.1186/s12877-022-03643-x

**Published:** 2022-12-05

**Authors:** Myung Chul Yoo, Chang Won Won, Yunsoo Soh

**Affiliations:** 1 Department of Physical Medicine and Rehabilitation, Kyung Hee University Medical Center, 23 Kyungheedae-ro, Dongdaemoon-gu, Seoul, 02447 Republic of Korea; 2 Department of Medicine, Graduate School, Kyung Hee University, Seoul, Republic of Korea; 3 Department of Family Medicine, Kyung Hee University Medical Center, 23 Kyungheedae-ro, Dongdaemoon-gu, Seoul, 02447 South Korea

**Keywords:** Aging, Body fat, Central obesity, Obesity, Sarcopenia

## Abstract

**Background:**

Age-related obesity and body composition changes include loss of muscle mass and increased body fat. This study aimed to investigate sex differences in the impact of sarcopenia, defined by the Asian Working Group for Sarcopenia (AWGS), on obesity in Korean older adults.

**Methods:**

In this 2-year longitudinal study, 3014 participants were excluded based on AWGS sarcopenia parameters (if any one of the sarcopenic parameter criteria was satisfied), including low handgrip strength (HGS), low appendicular skeletal muscle mass index (ASMI), and low short physical performance battery (SPPB). A total of 926 non-sarcopenic participants were recruited for the study. The obese and non-obese groups were compared according to the sarcopenia parameters. The following variables were selected for obesity analysis: body mass index (BMI), waist circumference (WC), and body fat percentage. Unadjusted and fully adjusted logistic regression analyses were performed for each variable to predict sarcopenia and sarcopenic obesity according to sex.

**Results:**

Among the sarcopenia parameters, reduction in ASMI was significantly lower in the obese group with high WC and percentage of body fat (PBF) in both men and women (*P* < 0.01). Multivariable analysis revealed that different obesity parameters were associated with AWGS criteria: women in the high BMI group presented significantly lower ASMI and sarcopenia (ASMI, OR = 0.289, 95% CI = 0.174-0.480; sarcopenia, OR = 0.152, 95% CI = 0.048-0.483). Women in the high WC group had significantly lower ASMI and sarcopenia (ASMI, OR = 0.307, 95% CI = 0.189-0.500; sarcopenia, OR = 0.262, 95% CI = 0.106-0.649). Women in the high PBF group had a lower incidence of sarcopenia (OR = 0.214, 95% CI = 0.068-0.278).

**Conclusions:**

Our study identified that high BMI had a protective effect on the reduction of muscle mass in men and women. However, obesity parameters including BMI, WC, and PBF were positively correlated with a lower incidence of sarcopenia only in women. Obesity in older women may have a protective effect in reducing ASMI and the incidence of sarcopenia.

## Introduction

Obesity, a chronic disease, is one of the most critical threats to health, including metabolic disorders that cause various medical problems and premature death. Therefore, its prevention and management are important. The prevalence of obesity in old age is increasing with socioeconomic development [1]. In Korea, the prevalence of obesity, defined as a body mass index (BMI) over 25 kg/m2, steadily increased from 35.6% in men and 23.9% in women in 2009 to 45.4% in men and 26.5% in women in 2018 [2]. Obesity increases the risk of chronic diseases such as hypertension, dyslipidemia, type 2 diabetes, and coronary artery disease [3]. Moreover, obesity in old age increases the risk of some cancer diseases and musculoskeletal problems, causing a reduction in physical function and quality of life [4]. BMI increases in the elderly because the height decreases due to decreased bone mass density, vertebral compression fracture, degeneration of the intervertebral disc, and kyphosis, leading to high BMI [5]. Other causes of increased BMI in the elderly are weight gain due to decreased physical activity and a decrease in baseline metabolic rate [6].

As hormone levels change with aging, the proportion of fat increases, lean components such as muscles and bones decrease, and body composition may change [7]. In addition, even if the amount of fat remains unchanged, it may be redistributed, with increases in the proportion of visceral obesity in the internal organs relative to subcutaneous fat, resulting in decreased overall strength and functionality [7, 8]. Waist circumference (WC) is used to evaluate body fat distribution and abdominal obesity [9]. The diagnostic criteria for central or abdominal obesity are 90 cm for men and 85 cm or more for women, and they are also at high risk for metabolic syndrome. Previous results have suggested that individuals who are overweight and obese have an increased risk of developing vitamin D deficiency, which is associated with decreased muscle performance [10]. In addition, low vitamin D correlates with impaired muscle function in postmenopausal women, and its combination with overweight further enhances muscle loss [10].

Sarcopenia is caused by skeletal muscle loss with aging [11]. Sarcopenia causes a decrease in skeletal muscle mass and a decrease in muscle strength or decreased physical function, resulting in disability and even mortality [12, 13]. A recent study found that the prevalence of sarcopenia in South Korea is approximately 21.3% in men and 13.8% in women aged 70-84 years [14], although it should be noted that rates of sarcopenia depend on the diagnostic method used. It is characterized by a loss of muscle strength associated with a decrease in muscle mass or quality, as assessed by lumbar muscle cross-sectional area via dual energy X-ray absorptiometry, computed tomography, or magnetic resonance imaging [15]. Bioelectrical impedance analysis, an inexpensive and readily available tool, can also be used to estimate total muscle mass. Additionally, in the context of ultrasound-based methodologies, shear wave elastic methods have recently been introduced that allow detection of abnormal muscle stiffness for the diagnosis of sarcopenia [16].

Previous studies have been conducted on an association between obesity and sarcopenia in the elderly. Although obesity is associated with premature death in adults, there is some report about the beneficial effect of obesity on lifespan in the elderly [17]. In our previous cross-sectional study of 1827 elderly Koreans, we found a protective effect of high BMI on sarcopenia in the elderly [18]; in particular, central obesity with high WC showed a low prevalence of sarcopenia. Adipose tissue, the primary site for storing and metabolizing sex hormones, is the main source of estrogen, and abdominal fat in women stores high levels of sex hormones and positively affects skeletal muscle mass [18]. However, these studies were cross-sectional, and there was a limitation in analyzing the causal effect of obesity on sarcopenia over time. In this 2-year longitudinal study, we evaluated the effect of obesity on the incidence of sarcopenia in elderly individuals without sarcopenia. This study also aimed to investigate the sex differences in the impact of obesity on sarcopenia, defined by the Asian Working Group for Sarcopenia (AWGS) 2019 guideline, in non-sarcopenic community-dwelling Korean older adults using data from the Korean Frailty and Aging Cohort Study (KFACS).

## Methods

### Data and study population

**Fig. 1:**
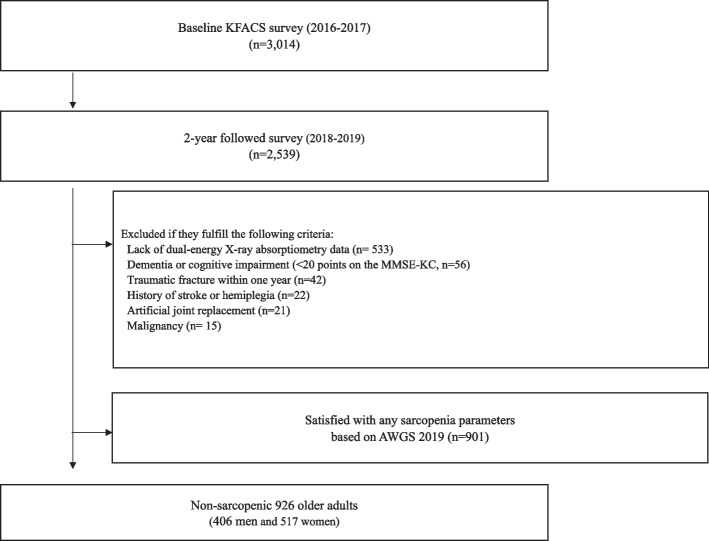
Flow chart of the participant recruitment process. KFACS, Korean Frailty and Aging Cohort Study; MMSE-KC, Mini-Mental Status Examination in the Korean version of the CERAD assessment packet

We used data from the KFACS gathered from 2016 to 2019 to investigate the 2-year longitudinal association between obesity and sarcopenia. Community-dwelling older adults aged 70–84 years were recruited in 2016 and 2017 at baseline across South Korea. A multicenter study was performed in eight medical and two public health centers. Body composition was assessed using bioelectrical impedance analysis (BIA) in two health centers and dual-energy X-ray absorptiometry (DXA) in eight hospital centers. Of the 3014 participants, 2539 returned to the survey 2 years later. Among them, 2006 participants completed the dual-energy X-ray absorptiometry (DEXA). Participants with hemiplegia, dementia, cognitive impairment (MMSE-KC < 25), blindness, malignancy, a traumatic fracture within 1 year, history of artificial joint replacement, incomplete data, and inability to complete physical tests were excluded. Subjects who met any of the criteria of low appendicular skeletal muscle mass (ASM), low muscle strength, or low physical performance according to the AWGS 2019 diagnostic criteria were also excluded (Fig. 1).

Demographic information and medical history were included in the analysis, such as age, sex, whether the family lives together, number of medications, income per month, location of residence (rural or urban), body measurements, chronic comorbidities, smoking, and alcohol status. Participants who drank alcohol at least once a week and smoked more than one cigarette per week were defined as drinkers and smokers. We mesaured the changes in reduction of sarcopenia parameters according to obesity including appendicular skeletal muscle mass index (ASMI), handgrip-strength (HGS), and short physical performance battery (SPPB). The study data protocol was approved by the Institutional Review Board (IRB) of the Clinical Research Ethics Committee of Kyung Hee University Medical Center (IRB number: 2015-12-103), and all participants provided written informed consent.

### Definition of disease status

#### Obesity

Obesity was classified according to body mass index (BMI), WC, and percentage of body fat (PBF). Body mass index (BMI) was calculated as body weight (kg) divided by height squared (m^2^). The obesity group was defined as having a BMI ≥ 25 kg/m^2^ according to the Asia-Pacific criteria of the World Health Organization guidelines [19]. Central obesity was defined as WC ≥ 90 cm in men and ≥ 85 cm in women, according to the definition of the Korean Society for the Study of Obesity (KSSO) [3]. WC was measured horizontally halfway between the superior iliac crest and the lower margin of the 12th rib. The percentage of body fat (PBF) was measured by DEXA, and obesity was defined as > 27% in men and > 38% in women [20, 21].

#### Sarcopenia

Sarcopenia was evaluated according to the AWGS 2019 diagnostic criteria [22]. Subjects with low appendicular skeletal muscle mass (ASM) and either low physical performance or muscle strength were defined as having sarcopenia.

1. Low muscle mass: DEXA was used to measure the ASM index, which was calculated for comparing appendicular muscle mass according to heights (ASM/height^2^: cutoff values- Men: < 7.0 kg/m2, Women: < 5.4 kg/m2). Of the eight study centers, four centers used Lunar (GE Healthcare, Madison, WI), and four used Hologic (Hologic Inc., Bedford, MA) DXA systems.
2. Low muscle strength: Hand grip strength (HGS) was measured using a hand dynamometer (K.K.5401; Takei Scientific Instruments Co. Ltd., Tokyo, Japan). Handgrip strength (kg) of each hand was measured twice, with the arms extended in a standing position. The participants held the handle with full force for 3 s, and the maximum of the four grip strength measurements was used in the analysis [23]. The cutoff values for low handgrip strength were < 28 kg for men and < 18 kg for women. (3) Low physical performance: Physical performance was assessed using the Short Physical Performance Battery (SPPB). The SPPB includes standing balance tests, 4 m gait speed, and five times chair stand test. Each test was assigned a score of 0 to 4 based on the normative scores obtained from the Established Population for Epidemiologic Studies of the Elderly, with a total score of 0 to 12. The 5CST is the time taken to stand up and sit down five times from a straight-backed armchair as quickly as possible without using arms folded across the chest [15]. Participants who could not complete the chair stand test (could not rise without using the arms or could not complete five stands) were classified as “fail” With a total of 12 points, low physical performance was defined as ≤9 points.

### Statistical analysis

Continuous and categorical variables were compared using Pearson’s chi-square and t-test, respectively. Unadjusted and fully adjusted analyses were performed using logistic regression models, and odds ratios (ORs) and 95% confidence intervals (CI) were calculated. Each analysis was fully adjusted for potential confounding variables such as age, hypertension, dyslipidemia, osteoarthritis, osteoporosis, diabetes mellitus, smoking history, alcohol history, location of residence, family living together, number of medications, and MMSE-KC were performed using logistic regression models, and odds ratios (ORs) and 95% confidence intervals (CI) were calculated. All statistical analyses were performed using the Statistical Package for Social Sciences (version 25.0; SPSS Inc., Chicago, Illinois, USA); *P* < 0.05 was defined as statistically significant.

## Results

The baseline characteristics of the participants are presented in Table 1. Finally, this study included 925 non-sarcopenic participants (406 men and 519 women). Among the 925 participants, 406 (43.8%) were men, and 519 (56.2%) were women. The prevalence of obesity, defined by BMI and PBF, was not significantly different between the sexes. However, the prevalence of central obesity was significantly higher in women than in men (men: 53.7% vs. women: 65.1%, *p* < 0.01). MMSE-KC, chronic comorbidities, and other socioeconomic characteristics, including family lives together, income per month, years of education, location of residence, and smoking habits, were significantly different between the sexes (Table 1).

**Table 1:** Baseline characteristics of the subjects according to sex

	Men (***n*** = 406)	Women (***n*** = 519)	***P***-value
Age (years)	76.3 ± 3.5	74.9 ± 3.6	0.53
BMI (kg/cm^2^)	25.2 ± 2.4	25.3 ± 2.7	0.47
Waist circumference (cm)	90.3 ± 7.6	87.6 ± 7.9	< 0.01*
PBF (%)	25.9 ± 5.3	36.6 ± 5.6	< 0.01*
Obesity by BMI			
Normal (BMI < 25 kg/cm^2^)	204 (50.2)	253 (48.7)	0.691
Obese (BMI ≥25 kg/cm^2^)	202 (49.8)	266 (51.3)	
Central obesity by WC			
No (men < 90, women < 85)	188 (46.3)	181 (34.9)	< 0.01*
Yes (men ≥90, women ≥85)	218 (53.7)	338 (65.1)	
Obesity by PBF			
No (men < 27, women < 38)	231 (56.9)	286 (55.1)	0.594
Yes (men ≥27, women ≥38)	175 (43.1)	233 (44.9)	
Family lives together (%)			
Together	372 (91.6)	261 (50.3)	< 0.01*
Alone	34 (8.4)	258 (49.7)	
Income per month			
More than 3	109 (26.8)	66 (12.7)	< 0.01*
(Korean million won^†^, %)			
1–3	166 (40.9)	189 (36.4)	
Less than 1	131 (32.3)	264 (50.9)	
Education years			
More than 13	114 (28.1)	42 (8.1)	< 0.01*
6–12	180 (44.3)	184 (35.5)	
Less than 6	112 (27.6)	293 (56.5)	
Location of residence (%)			
Urban	323 (79.9)	297 (86.2)	< 0.01*
Rural	81 (20.0)	71 (13.8)	
Current smoker (%)	40 (9.9)	2 (0.4)	< 0.01*
Alcohol use (%)	287 (70.7)	338 (65.1)	0.77
Hypertension (%)	209 (51.5)	314 (60.5)	< 0.01*
Dyslipidemia (%)	110 (27.1)	214 (41.2)	< 0.01*
Diabetes mellitus (%)	91 (22.4)	90 (17.3)	0.055
Depression (%)	6 (1.5)	3 (0.6)	0.192
Knee OA (%)	45 (11.1)	166 (32.0)	< 0.01*
Osteoporosis (%)	10 (2.5)	112 (21.6)	< 0.01*
MMSE-KC	26.7 ± 2.4	25.8 ± 2.8	< 0.01*

*BMI* body mass index, *WC* waist circumference, *PBF* percentage of body fat, *OA* osteoarthritis, *MMSE-KC* Mini-Mental Status Examination-Korean version

^*^P < 0.01

^†^1 million Korean won is approximately 800 US dollars

**Fig. 2:**
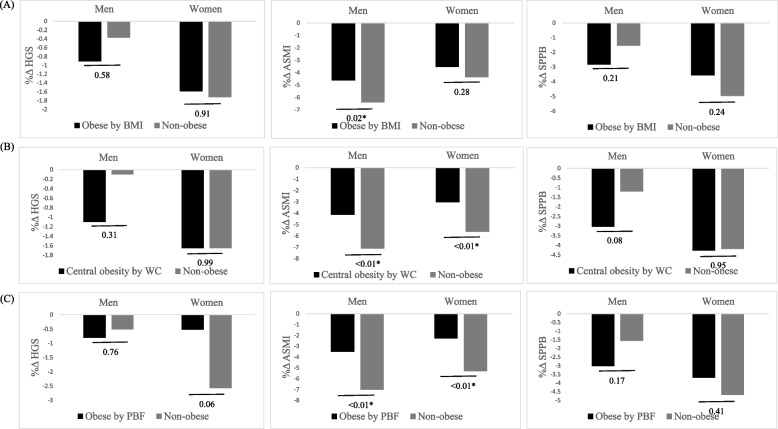
Changes in reduction of sarcopenia parameters according to obesity. Black rectangles and grey rectangles indicate obesity and non-obese, respectively. Relative percentage change of appendicular skeletal muscle mass index (ASM), handgrip-strength (HGS), and short physical performance battery (SPPB). **A** Obesity defined by body mass index (BMI, ≥ 25 kg/m2). **B** Central obesity defined by waist circumference (WC, ≥ 90 cm for men and ≥ 85 cm for women). **C** Obesity defined by the percentage of body fat (PBF, ≥ 28% for men and ≥ 34% for women)

Table 2 shows the 2-year longitudinal effect of obesity on the changes in sarcopenia parameters according to sex. All sarcopenia parameters deteriorated after 2 years of follow-up. Obesity defined by BMI did not affect changes (Δ) in HGS, ASMI, and SPPB in either sex. In contrast, central obesity by WC and general obesity by PBF were associated with a lower decrease in ASMI than non-obesity in both sexes. Additionally, relative percentage change of ASMI, HGS, and SPPB were evaluated. Obesity defined by BMI, WC and PBF affected relative changes (Δ) in ASMI in either sex execpt women in BMI (Fig. 2).

**Table 2:** Two-year effect of obesity on sarcopenia parameters according to sex

	Men			Women		
	BMI ≥ 25 (*n* = 202)	BMI < 25 (*n* = 204)	*P*-value	BMI ≥25 (*n* = 266)	BMI < 25 (*n* = 253)	*P*-value
Δ HGS, kg (mean ± SD)	− 0.33 ± 3.56	− 0.22 ± 3.13	0.747	−0.45 ± 2.93	−0.41 ± 2.60	0.852
Δ ASMI, kg/m^2^ (mean ± SD)	− 0.38 ± 0.62	− 0.48 ± 0.59	0.084	− 0.24 ± 0.56	−0.27 ± 0.56	0.507
Δ SPPB (mean ± SD)	−0.35 ± 1.26	−0.20 ± 1.09	0.187	−0.43 ± 1.44	−0.58 ± 1.56	0.249
	WC ≥90 (*n* = 218)	WC < 90 (*n* = 188)	*P* value	WC ≥85 (*n* = 338)	WC < 85 (*n* = 181)	*P* value
Δ HGS, kg (mean ± SD)	−0.09 ± 3.18	−0.44 ± 3.48	0.302	−0.41 ± 2.63	−0.44 ± 2.84	0.890
Δ ASMI, kg/m^2^ (mean ± SD)	−0.33 ± 0.59	−0.54 ± 0.62	< 0.001**	−0.20 ± 0.54	−0.35 ± 0.57	0.04*
Δ SPPB (mean ± SD)	−0.37 ± 1.24	−0.16 ± 1.10	0.072	−0.51 ± 1.48	−0.49 ± 1.55	0.902
	PBF ≥ 28 (*n* = 175)	PBF < 28 (*n* = 231)	*P* value	PBF ≥ 34 (*n* = 233)	PBF < 34 (*n* = 286)	*P* value
Δ HGS, kg (mean ± SD)	−0.30 ± 3.46	−0.26 ± 3.27	0.907	−0.22 ± 2.91	−0.6 ± 2.64	0.125
Δ ASMI, kg/m^2^ (mean ± SD)	−0.27 ± 0.52	−0.55 ± 0.65	< 0.001**	−0.15 ± 0.47	−0.70 ± 0.67	< 0.001**
Δ SPPB (mean ± SD)	−0.20 ± 1.10	−0.27 ± 0.52	0.147	−0.44 ± 1.45	−0.56 ± 1.54	0.374

Cut-off point of obesity: BMI ≥ 25 kg/m^2^; WC ≥ 90 cm for men and ≥ 85 cm for women; PBF ≥ 28% for men and ≥ 34% for women

*BMI* body mass index, *WC* waist circumference, *PBF* percentage of body fat, *HGS* hand grip strength, *ASMI* appendicular skeletal muscle mass index, *SPPB* short physical performance battery

^*^P < 0.05

^**^P < 0.001

Table 3 shows the 2-year longitudinal effect of obesity defined as BMI ≥ 25 on the criteria of sarcopenia parameters according to sex. Both the unadjusted and fully adjusted models showed a lower incidence of low ASMI in the high BMI group in both sexes during the 2-years follow-up period. (odds ratio [OR] = 0.344, 95% confidence interval [CI] = 0.213–0.555 in men; OR = 0.289, 95% CI = 0.174–0.480 in women). However, as for sarcopenia, in only “women”, high BMI was associated with lower incident sarcopenia (OR = 0.152, 95% CI =0.048–0.483).

**Table 3:** Logistic regression analysis of obesity defined by BMI for sarcopenia parameters according to sex

Characteristic^a^	Unadjusted model		Fully adjusted model	
	Men	Women	Men	Women
	OR (95% CI)	OR (95% CI)	OR (95% CI)	OR (95% CI)
**Muscle strength** Low HGS	1.856 (0.888-3.882)	0.797 (0.449-1.416)	1.891 (0.836-4.274)	0.895 (0.489-1.639)
**Muscle mass** Low ASMI	0.364** (0.235-0.563)	0.326** (0.204-0.521)	0.344** (0.213-0.555)	0.289** (0.174-0.480)
**Physical performance** Low SPPB	1.363 (0.689-2.695)	0.994 (0.629-1.573)	1.515 (0.731-3.140)	0.983 (0.604 -1.600)
Sarcopenia^b^	0.726 (0.325-1.621)	0.160** (0.054-0.472)	0.734 (0.300-1.795)	0.152** (0.048-0.483)

The fully adjusted model was adjusted for age, hypertension, dyslipidemia, osteoarthritis, osteoporosis, diabetes mellitus, smoking history, alcohol history, location of residence, family group, number of medications, and the MMSE-KC score

Obesity defined as BMI ≥ 25 kg/m2

*PBF* percentage of body fat, *OR* odds ratio, *CI* confidence interval, *HGS* hand grip strength, *ASMI* appendicular skeletal muscle mass index, *SPPB* short physical performance battery

^a^Low HGS, < 28 kg for males and < 18 kg for females; low ASMI, < 7.0 kg/m2 for males and < 5.4 kg/m2 for females; low SPPB, score ≤ 9 for both sexes

^b^Sarcopenia: low ASMI (< 7.0 kg/m2 for men and < 5.4 kg/m2 for women) and either low HGS (< 28 kg for men and < 18 kg for women) or low physical performance (SPPB score ≤ 9 for both sexes)

^*^P < 0.05

^**^P < 0.001

Table 4 shows the 2-year longitudinal effect of central obesity defined by WC on the sarcopenia parameters according to sex criteria. Similar to BMI, central obesity was associated with the incidence of low ASMI in both men and women, but it was associated with a low incidence of sarcopenia in only “women” (OR = 0.262, 95% CI = 0.106–0.649).

**Table 4:** Logistic regression analysis of central obesity defined by WC for sarcopenia parameters according to sex

Characteristic^a^	Unadjusted model		Fully adjusted model	
	Male OR (95% CI)	Female OR (95% CI)	Male OR (95% CI)	Female OR (95% CI)
**Muscle strength** Low HGS	1.187 (0.578-2.437)	0.704 (0.393-1.260)	1.130 (0.506-2.526)	0.748 (0.400-1.399)
**Muscle mass** Low ASMI	0.463** (0.303-0.708)	0.326** (0.208-0.508)	0.446** (0.281-0.709)	0.307** (0.189-0.500)
**Physical performance** Low SPPB	1.295 (0.651-2.574)	1.256 (0.766-2.059)	1.434 (0.691-2.975)	1.252 (0.173 -2.128)
**Sarcopenia**^b^	0.517 (0.229-1.168)	0.264** (0.115-0.605)	0.490 (0.194 -1.240)	0.262** (0.106-0.649)

The fully adjusted model was adjusted for age, hypertension, dyslipidemia, osteoarthritis, osteoporosis, diabetes mellitus, smoking history, alcohol history, location of residence, family group, number of medications, and the MMSE-KC score

Central obesity defined by waist circumference ≥ 90 cm for men and ≥ 85 cm for women

*WC* waist circumference, *OR* odds ratio, *CI* confidence interval, *HGS* hand grip strength, *ASMI* appendicular skeletal muscle mass index, *SPPB* short physical performance battery

^a^Low HGS, < 28 kg for males and < 18 kg for females; low ASMI, < 7.0 kg/m2 for males and < 5.4 kg/m2 for females; low SPPB, score ≤ 9 for both sexes

^b^Sarcopenia: low ASMI (< 7.0 kg/m2 for men and < 5.4 kg/m2 for women) and either low HGS (< 28 kg for men and < 18 kg for women) or low physical performance (SPPB score ≤ 9 for both sexes)

^*^P < 0.05

^**^P < 0.001

Table 5 shows the logistic regression analysis results that predicted sarcopenia and its parameters based on high PBF by sex. In the fully adjusted model, high PBF was associated with a lower sarcopenia incidence only in “women” (OR = 0.214, 95% CI = 0.068–0.678).

**Table 5:** Logistic regression analysis of obesity defined by PBF for sarcopenia parameters according to sex

Characteristic^a^	Unadjusted model		Fully adjusted model	
	Male OR (95% CI)	Female OR (95% CI)	Male OR (95% CI)	Female OR (95% CI)
**Muscle strength** Low HGS	0.890 (0.498-1.588)	1.888 (0.919-3.880)	2.001 (0.892-4.489)	1.078 (0.580-2.006)
**Muscle mass** Low ASMI	0.720 (0.470-1.103)	0.726 (0.466-1.133)	0.732 (0.457-1.173)	0.678 (0.415-1.106)
**Physical performance** Low SPPB	1.624 (0.824-3.202)	1.086 (0.686-1.724)	1.824 (0.865-3.845)	1.189 (0.723 -1.954)
**Sarcopenia**^b^	1.587 (0.715-3.523)	0.210** (0.071-0.617)	1.796 (0.716 -4.503)	0.214** (0.068-0.678)

The fully adjusted model was adjusted for age, hypertension, dyslipidemia, osteoarthritis, osteoporosis, diabetes mellitus, smoking history, alcohol history, location of residence, family group, number of medications, and the MMSE-KC score

Obesity defined by PBF ≥ 28% for men and ≥ 34% for women

*OR* odds ratio, *CI* confidence interval, *HGS* hand grip strength, *ASMI* appendicular skeletal muscle mass index, *SPPB* short physical performance battery

^a^Low HGS, < 28 kg for males and < 18 kg for females; low ASMI, < 7.0 kg/m2 for males and < 5.4 kg/m2 for females; low SPPB, score ≤ 9 for both sexes

^b^Sarcopenia: low ASMI (< 7.0 kg/m2 for men and < 5.4 kg/m2 for women) and either low HGS (< 28 kg for men and < 18 kg for women) or low physical performance (SPPB score ≤ 9 for both sexes)

^*^P < 0.05

^**^P < 0.001

## Discussion

This study investigated the association between BMI, WC, and PBF, which are several criteria for obesity and sarcopenia, according to sex over 2 years. In the model adjusted for confounding variables, obesity defined by a high BMI was associated with a protective effect on low ASMI. We also found that central obesity based on WC and high PBF was only associated with a lower incidence of sarcopenia in women. In our previous cross-sectional study, a high BMI > 25 was associated with a low prevalence of sarcopenia and low ASMI, as defined by the AWGS criteria in both sexes, and central obesity was associated with a lower prevalence of sarcopenia in women [18]. A limitation of this previous study is that it was difficult to clarify the causal relationship between obesity and sarcopenia. However, in this longitudinal study, by observing time-dependent changes in sarcopenic parameters over 2 years, it was possible to investigate the relationship between various obesity variables and sarcopenia incidence according to sex. With aging, a decrease in muscle mass, physical function, and muscle strength could be observed, and it was found that a high BMI had a protective effect on the reduction of ASMI in both sexes. In addition, high BMI, WC, and PBF were found to be independent protective factors for the incidence of sarcopenia only in women.

Generally, with aging, fat content increases, and muscle mass decreases by 0.5-1.0% per year [24]. Changes in body composition are thought to be due to a decrease in skeletal muscle mass and an increase in body fat percentage due to physiological changes in energy metabolism hormones during aging [8]. However, the proportion of muscle mass also tends to be high in patients with high BMI, and even in patients with metabolic syndrome, there is a positive relationship between high BMI and muscle mass [25]. Previous studies also reported that skeletal mass decreased with aging in both sexes, but the skeletal mass index positively correlated with increasing BMI [26].

Research is ongoing on how obesity may affect the progression of sarcopenia in old age and sex. A vicious cycle that induces inflammation in a large amount of adipose tissue and skeletal muscle can lead to the onset and exacerbation of sarcopenia. In particular, the combination of reduced lean body mass and increased visceral fat with aging may accelerate skeletal muscle weakness in elderly individuals. A combination of reduced lean body mass and increased visceral fat is also associated with decreased physical function, increased risk of disability, worsening hospitalization, increased morbidity, and premature mortality [27]. An increase in visceral fat can lead to systemic inflammation and insulin resistance, which can in turn lead to low muscle mass and sarcopenia in the elderly [28]. In addition, decreased movement due to loss of skeletal muscle function accelerates muscle fat infiltration, which is closely related to physical inactivity [29]. However, some studies have suggested the potential paradox that obesity might protect skeletal muscle mass in old age [30]. High BMI was significantly associated with stronger antigravity muscles and increased lower extremity skeletal muscle size as measured by CT; chronic overload causes hypertrophy in the lower extremity muscles to help maintain upright and balanced postures [31, 32]. A study of the risk factors for sarcopenia in adults living in nursing homes found that low BMI was a predictor of sarcopenia [33]. Additionally, previous studies have shown lower mortality in elderly individuals with normal or overweight BMI (< 30 kg/m2) and high BMI has been shown to have “survival benefits” [34, 35]. Our study excluded elderly individuals who could not complete the physical examination due to movement limitations such as hemiplegia, cognitive impairment, blindness, and inability to complete physical tests. Subjects who met any of the criteria of normal ASM, muscle strength, or physical performance according to the AWGS 2019 diagnostic criteria were included, as shown in Fig. 1. Among our elderly study subjects, the obese group may have less muscle mass loss than the group with relatively less mobility due to the greater load required for muscle movement in the former group. Our study also showed that muscle loss was low in normal elderly with high BMI without sarcopenia after a relatively two-year follow-up time. In particular, there was less odds ratio to be afflicted with sarcopenia in women. In addition, BMI alone may not fully capture changes in fat and lean mass in the elderly, suggesting the need to comprehensively consider WC and PBF together.

BMI is primarily used as a simple indicator of obesity and is calculated by dividing body weight by the square of height [36]. However, several studies have criticized the use of BMI indicators to provide an incomplete understanding of actual body composition, particularly body fat distribution [37]. Specifically, BMI does not consider the loss of muscle mass and increased fat with age. In addition, conventional BMI cutoff values or arbitrarily assigned body fat percentage criteria to define overweight and obesity may misclassify the elderly and underestimate the prevalence of excess body fat [38]. Therefore, it has also been argued that the BMI used to define obesity does not appear to be an appropriate indicator of obesity in the elderly [39, 40]. Previous studies suggesting the ‘obesity paradox’ due to the limitations of BMI have shown that BMI is not obese. This remains controversial because it was evaluated only as a parameter.

WC, an obesity parameter, estimates central obesity and is a better predictor of cardiac metabolic morbidity and premature mortality than BMI, especially in people and women with a low BMI [41, 42]. In our study, WC was the most predictive indicator of obesity for physical and functional limitations in women, which is consistent with the results of previous studies. Our results showed that loss of visceral fat and lean mass might be more important than BMI in determining obesity-related health risks in the elderly.

The mechanism by which obesity in older adults appears to protect muscle mass is unclear, and it is difficult to determine the exact cause and effect due to the complex interplay between obesity and sarcopenia. Several hypotheses could explain this finding. First, skeletal muscle stimulation was increased as a higher level of muscle mass was observed in the obese group. Over time, there is less loss of muscle mass owing to the greater load required for exercise [27]. Second, because BMI is determined by height and weight, it cannot distinguish lean mass from body fat. Therefore, even non-sarcopenic elderly individuals with more muscle without fatty degeneration have a high BMI, so they have the disadvantage of being classified into the obesity group. Therefore, it is possible that a high BMI in the elderly could not distinguish obesity from high muscle mass during body composition changes. In addition, a result showed a relatively poor correlation between PBF and BMI, and BMI correlated better with lean body mass than with fat mass, supporting this hypothesis.

A notable result from this study is that both WC and PBF, closely related to the amount of visceral fat in women, play a protective role in sarcopenia. Increased leptin production by adipocytes contributes to ectopic fat deposition in the muscle, which reduces muscle quality and strength [43–45]. Adipose fat tissue from obese individuals has high levels of tumor necrosis factor-alpha (TNF-α), which promotes the production and secretion of several pro-inflammatory cytokines. These pro-inflammatory cytokines promote catabolic pathways that promote muscle wasting and ultimately impair muscle function restoration. In contrast to these metabolic pathways, Chen et al. reported that central obesity is associated with a lower risk of muscle mass loss in menopausal women, which is consistent with our findings [46]. Because abdominal fat stores high concentrations of sex hormones and positively affects skeletal muscle mass [43, 47]. Adipose tissue, the main site for storing and metabolizing sex hormones, is the main source of estrogen. Healthy adipocytes secrete adiponectin, an anti-inflammatory and insulin sensitizer that is positively associated with muscle cells [48]. This could explain the protective effect of sarcopenia in women compared to that in men, as found in studies of the interaction between adipocytes and muscle cells. These findings are consistent with those of this longitudinal study as well as the cross-sectional study we previously reported.

This study had several limitations. First, due to East Asian characteristics, the number of patients with severe obesity (BMI > 30 was relatively small (*n* = 48, 5.1% of the total participants); therefore, our results might not be adequately reflected in the case of severe obesity. Second, in the survey conducted 2 years later, the follow-up loss was 475 persons in this study, which was confirmed to be 15.7% of the initial 3014 persons. However, this was similar to or even lower than in previous cohort studies. Third, this study might not apply to other populations because it was conducted in a single race, the Korean population. As body composition differs, studies on different populations are warranted.

## Conclusion

This was the first longitudinal cohort study to investigate the association between obesity and the component parameters of sarcopenia in non-sarcopenic elderly individuals. Our study identified that Korean elderly with obesity had a protective impact on the reduction of muscle mass in men and women. However, obesity parameters including BMI, WC, and PBF were positively correlated with a lower incidence of sarcopenia only in women. Obesity in older women may have a protective effect on reducing ASMI and the incidence of sarcopenia.

## Data Availability

Supporting data and data analysis materials are available from the corresponding author (Prof. Yunsoo Soh) upon request.
